# Neuron-Specific Fluorescence Reporter-Based Live Cell Tracing for Transdifferentiation of Mesenchymal Stem Cells into Neurons by Chemical Compound

**DOI:** 10.1155/2017/8452830

**Published:** 2017-07-20

**Authors:** Do Won Hwang, Hyun Woo Kwon, Jaeho Jang, Hee Jung Jung, Kwang Rok Kim, Dong Soo Lee

**Affiliations:** ^1^Department of Nuclear Medicine, Seoul National University College of Medicine, Seoul, Republic of Korea; ^2^Department of Molecular Medicine and Biopharmaceutical Sciences, Graduate School of Convergence Science and Technology and College of Medicine or College of Pharmacy, Seoul National University, Seoul, Republic of Korea; ^3^Department of Nuclear Medicine, Korea University Anam Hospital and Korea University College of Medicine, Seoul, Republic of Korea; ^4^Drug Discovery Division, Korea Research Institute of Chemical Technology, Daejeon, Republic of Korea

## Abstract

Although transdifferentiation of mesenchymal stem cells (MSCs) into neurons increases the possibility of therapeutic use of MSCs for neurodevelopmental disorders, the use of MSCs has the limitation on differentiation efficiency to neuronal lineage and lack of an easy method to monitor the transdifferentiation. In this study, using time-lapse live cell imaging, we assessed the neuronal differentiation of MSCs induced by a small molecule “NHPDQC (N-hydroxy-2-oxo-3-(3-phenylprophyl)-1,2-dihydroquinoxaline-6-carboxamide, C_18_H_17_N_3_O_3_).” Plasmid vector containing red fluorescence reporter genes under the control of the tubulin *α*1 (T*α*1) promoter (pT*α*1-DsRed2) traced the neuronal differentiation of MSCs. Two days after NHPDQC treatment, MSCs showed neuron-like phenotype with neurite outgrowth and high expression of neuron-specific markers in more than 95% cells. The fluorescence signals increased in the cytoplasm of pT*α*1-DsRed2-transfected MSCs after NHPDQC treatment. In vitro monitoring of MSCs along the time courses showed progressive increase of fluorescence till 30 h after treatment, corresponding with the increase in neurite length. We examined an efficient neuronal differentiation of MSCs by NHPDQC alone and monitored the temporal changes of neuronal differentiation by neuron-specific fluorescence reporter along time. This method would help further our understanding of the differentiation of MSCs to produce neurons by simple treatment of small molecule.

## 1. Background

Mesenchymal stem cells (MSCs) are nonhematopoietic stem cells in the bone marrow (BM), which are able to differentiate into mesodermal lineage cells including osteogenic, chondrogenic, adipogenic, and other mesenchymal lineage cells [1–4]. MSCs may be the best candidate for stem cell-based replacement therapy because they can be easily collected from humans and have relatively low immunogenicity [5–7].

Evidence for therapeutic effects of MSCs have accumulated in recent studies, showing that MSCs exert immunomodulatory effects by reducing proinflammatory activity and neuroprotection via secreting neurotrophic factors to prevent further neuronal injury [8–12]. Intrastriatal transplantation of GDNF- (glial cell line-derived neurotrophic factor-) treated MSCs improved behavior in movement impairment in a rat model of Parkinson's disease [13]. Transplantation of BM-derived MSCs through intraperitoneal injection delayed disease onset and increased life span in an amyotrophic lateral sclerosis (ALS) mouse model [14]. However, all these therapeutic effects could have been due to positive “nonneuronal effects” rather than direct neurogenesis from MSCs. Furthermore, the grafted MSCs might continuously proliferate in vivo and make tumors. The simple method described here efficiently makes MSCs differentiate to neurons and, importantly, allows the in vitro time-lapse live cell monitoring of this transdifferentiation of MSCs.

**Figure 1:**
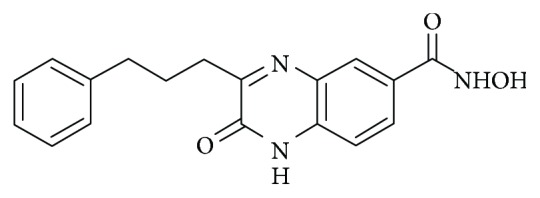
The quinoxaline-based small molecule, NHPDQC (N-hydroxy-2-oxo-3-(3-phenylprophyl)-1,2-dihydroquinoxaline-6-carboxamide), was structurally modified to induce neuronal differentiation of rat MSCs.

In our previous work, a new small molecule, N-hydroxy-2-oxo-3-(3-phenylprophyl)-1,2-dihydroquinoxaline-6-carboxamide, named as “NHPDQC” was reported to induce transdifferentiation of MSCs into neurons with high efficiency (Figure 1()). NHPDQC was proposed as potent neuronal inducer in view of neuron-specific gene expression and electrophysiological properties without cellular toxicity [15].

Neuron-specific promoter-regulated reporters have been widely used to trace in vivo characteristics of individual neurons [16–19]. Especially, this technique enables the monitoring of stem cell differentiation and the screening of small molecule candidate capable of inducing neuronal differentiation [20]. One previous study showed that glial fibrillary acidic protein (GFAP) promoter-driven fluorescence reporter was used to purify differentiated astrocytes from embryonal stem cells and sort out the astrocyte cell lineage from heterogeneous cell population [21]. Tubulin *α*1 (T*α*1) promoter has been used often as one of the promoters which are specific for neurons [22–26], and T*α*1 promoter-driven reporters can be used to trace early neuronal differentiation of stem cells along with the increase of T*α*1 promoter activity in the early phase of neuronal differentiation. As NHPDQC facilitates neuronal differentiation of MSCs in a short period of time, T*α*1 promoter-driven reporter system is appropriate for visualizing the effect of NHPDQC on MSCs over time.

In this study, we applied time-lapse live cell imaging to track the in vitro changes of neuronal differentiation of MSCs induced by NHPDQC using T*α*1 promoter-driven fluorescence reporter gene.

## 2. Methods

### 2.1. Neuronal Transdifferentiation of MSCs Using NHPDQC

MSCs were isolated from the femur bone marrow of male Fisher rats. The cells were then maintained in Dulbecco's modified Eagle's medium (DMEM; Invitrogen, Grand Island, NY, USA), containing 10% of fetal bovine serum (FBS; Invitrogen, Grand Island, NY, USA), along with 100 U/ml penicillin, 100 *μ*g/ml streptomycin, and 0.25 *μ*g/ml amphotericin B (Gibco, Grand Island, NY, USA). The nonadherent cells were removed after 48 h, and the adherent cells were washed with phosphate-buffered saline (PBS) and then cultured in fresh medium. The cultured cells were maintained for 12 to 20 passages during the experiment. The MSCs were seeded with the initial density of 4 × 10^5^ cells per 10 cm plate. The MSCs were treated with NHPDQC at a concentration of 20 *μ*M and 0.1% dimethyl sulfoxide (DMSO: Sigma-Aldrich, St. Louis, USA), followed by incubation in a standard incubator with 5% CO_2_ for 72 h. The MSCs in the control group were treated with the same amount of DMSO and incubated under the same conditions. The morphologies of the cells were detected by phase contrast microscopy.

### 2.2. Reverse Transcription Polymerase Chain Reaction (RT-PCR)

The total RNA of the MSCs was isolated using TRIzol reagent (Invitrogen, Grand Island, NY, USA) and was reverse-transcribed using a first-strand cDNA synthesis kit (Invitrogen, Grand Island, NY, USA). The sequences of forward and reverse primers were described in Table 1. The polymerase chain reaction (PCR) was performed for 30 cycles (denaturation at 94°C for 30 s, annealing at 56°C for 30 s, and extension at 72°C for 60 s).

### 2.3. Immunohistochemistry

Cells were fixed with 4% paraformaldehyde (PFA: Wako Pure Chemical, Osaka, Japan) for 20 min at room temperature. After blocking with normal serum, the cells were incubated in 0.1% Triton X-100 in PBS containing primary mouse antibody Tuj1 against brain-specific *β*III-tubulin (1 : 200 dilution: TU-20, Cell Signaling) and *β*-tubulin (1 : 50 dilution, D-10, Santa Cruz) for 24 h at 4°C. The cells were washed and incubated with Alexa Fluor 488-conjugated goat anti-mouse secondary antibodies (Invitrogen, Grand Island, NY, USA). Fluorescence images were obtained with a Carl Zeiss LSM 510 microscope.

### 2.4. Transfection of Fluorescent Reporter Gene

A plasmid construct with a T*α*1 promoter driving red fluorescent protein expression, pT*α*1-DsRed2, was kindly provided by Dr. Yoon K (Sungkyunkwan University, Seoul, Korea) [25]. The plasmid was transfected into MSCs by incubating for 2 h with Lipofectamine Plus (Invitrogen, Carlsbad, CA), diluted in OPTI-MEM medium (Gibco, Grand Island, NY). Subsequently, the cells were washed with PBS and cultured for 48 h in a serum-containing growth medium.

### 2.5. Confocal Microscopy and Live Cell Imaging

The cells were seeded on sterile cover slips in 24-well plates and cultured for 24 h. They were then fixed using 4% PFA under gentle shaking for 20 min, followed by washing with PBS. Transfer slides were prepared with a mounting solution containing 4′,6-diamidino-2-phenylindole dihydrochloride (DAPI) solution (Vector Laboratories Inc., Burlingame, CA, USA). A confocal laser scanning microscope (LSM 510; Carl Zeiss Inc., Thornwood, NY, USA) was used for fluorescence imaging: detection was carried out at a wavelength of 405 nm for DAPI and at 573 nm for DsRed2. The length of neurite growth in MSCs with neuronal transdifferentiation was manually measured on at least 3 acquired images. The cells were placed in an incubation chamber equipped with a time-lapse imaging system (Olympus IX81 microscope). Phase contrast and fluorescence images were obtained simultaneously, at 30 min intervals, until 72 h following treatment. Quantitative analysis for measuring neurite growth and fluorescence signal in the DMSO-treated cells and the NHPDQC-treated cells were performed by implemented software (METAMORPH 7.5.6, MDS Analytical Technologies, PA, USA). And confocal data was used for quantitative analysis using TissueFAXS2.0. After the samples were prescanned, the region of interest was automatically measured. Individual fluorescence signals from region of interest were detected using TissueFAXS2.0. Cell analysis software, TissueQuest, was used for analyzing total fluorescence intensity versus DAPI in the whole cell population (TissueGnostics, CA, USA).

### 2.6. Statistical Analysis

Continuous variables were tested using Student's *t*-test. Data were expressed as mean ± standard deviation, and *P* values smaller than 0.05 were considered significant.

## 3. Results

### 3.1. Morphological Changes of MSCs with NHPDQC Treatment

The NHPDQC was synthesized as 1-bromo-3-phenylpropane underwent coupling reaction with dimethyl oxalate, then ketoester cyclization with methyl 3,4-diaminobenzoate, and finally introduction of hydroxylamine using tetrahydropyranyloxyamine and trifluoroacetic acid, sequentially [15].

**Figure 2:**
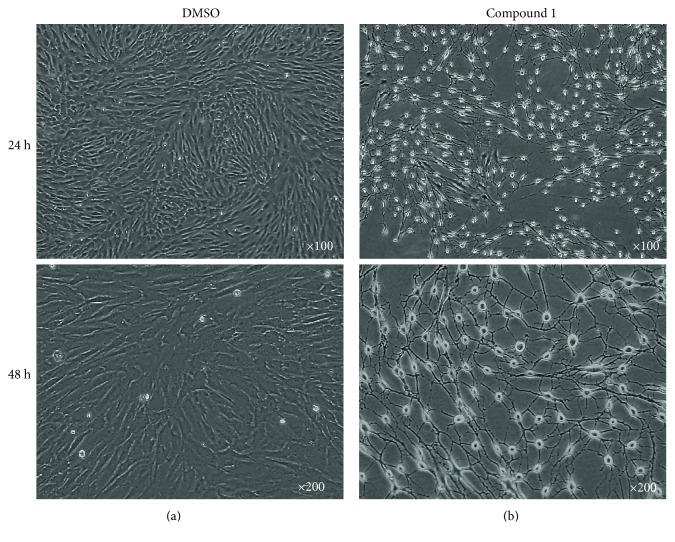
Morphological changes of (a) the DMSO-treated mesenchymal stem cells (MSCs) or (b) the NHPDQC-treated MSCs at 24 h (upper panel) and 48 h (lower panel) after treatment. MSCs treated with NHPDQC exhibited neurite growth, along with cell body retraction.

MSCs treated with DMSO alone as a control did not show any morphological changes (Figure 2(a)). In contrast, two days after incubation of MSCs with 20 *μ*M NHPDQC, most MSCs showed apparent neuron-like morphological changes, including a spindle-like retraction of the cell body along with the elongated neurite outgrowth (Figure 2(b)). On live cell microscope imaging, the NHPDQC-treated MSCs started into neuron-like differentiation within 24 h, and almost all of MSCs (approximately >95%) formed finally neuron-like phenotype at 48 h (Figure 2(b), lower panel). Whereas the total number of cells did not increase substantially in the NHPDQC-treated MSCs, the DMSO-treated control MSCs increased gradually in cell number until 48 h.

### 3.2. Evaluation of Neuronal-Specific Marker Expression in MSCs Treated with NHPDQC

**Figure 3:**
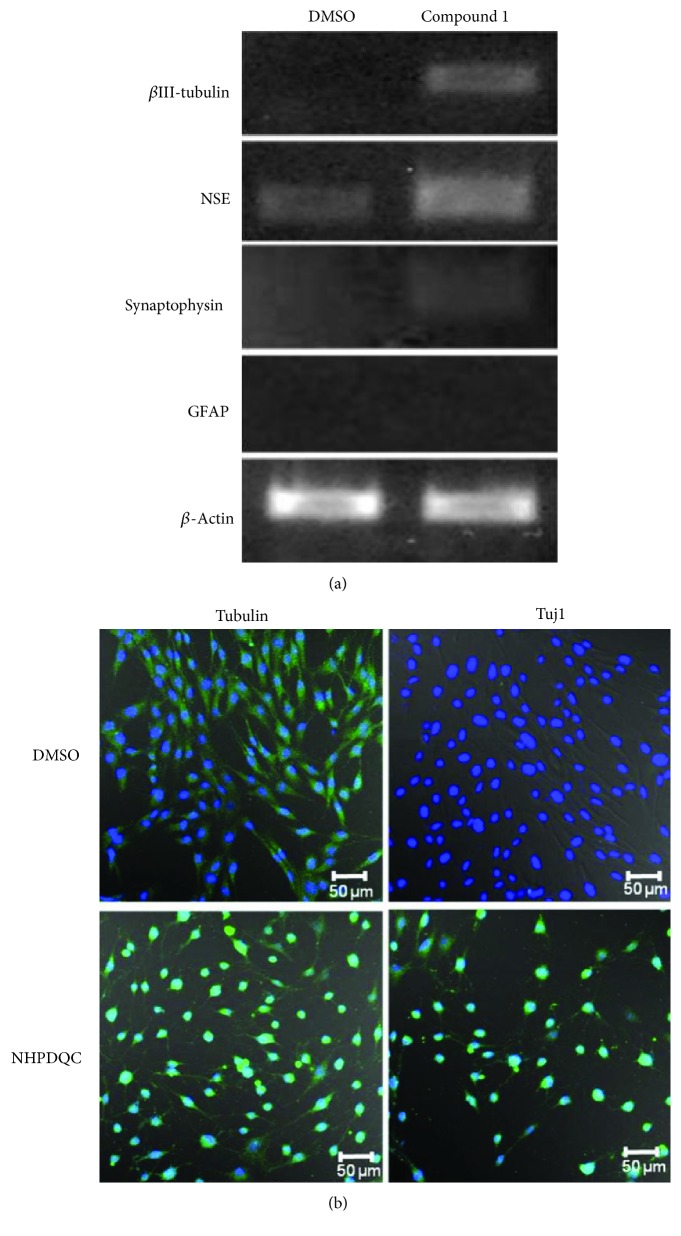
Neuronal differentiation of MSCs after treatment of NHPDQC. (a) Reverse transcription polymerase chain reaction (RT-PCR) results (left column: DMSO-treated group, right column: NHPDQC treatment group). Expression of the neuronal markers Tuj1 and NSE was increased in the MSCs at 48 h after treatment with NHPDQC. The RT-PCR results revealed that the expression of the presynaptic vesicle protein, synaptophysin, was increased slightly after 48 h of treatment with NHPDQC. Glial marker GFAP did not increase in both of undifferentiated and differentiated MSCs. (b) Immunofluorescence staining was performed in the DMSO-treated cell group and the group treated with NHPDQC. The results showed that Tuj1 expression was increased in the NHPDQC-treated group within 48 h. Green color: *β*-tubulin (upper panel), Tuj1 (lower panel); blue color: DAPI.

On RT-PCR analysis for neuron-specific gene expression at 48 h after NHPDQC treatment, early postmitotic neuronal marker (neuron-specific *β*III-tubulin), and other neuron-specific markers, NSE expressed significantly higher in MSCs (Figure 3(a)). In the MSCs treated with only DMSO as the control group, *β*III-tubulin expression was not detected, but NSE expression was detected scantly. Glial marker GFAP expression was not detected in either the treatment or control group. Expression of the presynaptic vesicle protein, synaptophysin, increased slightly in the NHPDQC-treated MSCs within the treatment group (Figure 3(a)). Immunofluorescence staining revealed that the cell shape was changed in the NHPDQC-treated cells using cytoskeleton protein. The NHPDQC treatment also increased the *β*III-tubulin expression in the cytoplasm of the MSCs (Figure 3(b)). These results demonstrated that NHPDQC triggered MSCs into early neuronal lineage within 48 hr postinduction in terms of immunophenotype with relevant markers.

### 3.3. In Vitro Transdifferentiation Imaging into Neurons in Living MSCs

**Figure 4:**
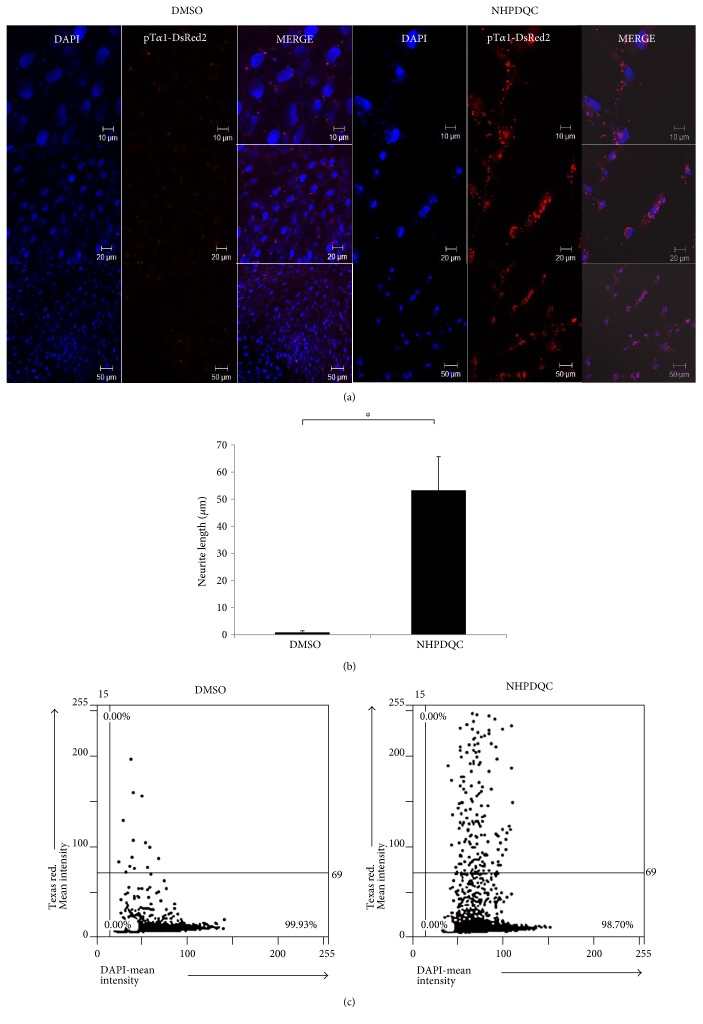
Enhanced fluorescence signals after neuronal differentiation of pT*α*1-DsRed2-transfected MSCs by NHPDQC. (a) Confocal microscopic data showed that T*α*1 promoter-regulated RFP reporter activity was increased in the NHPDQC-treated group within 48 h (blue color: DAPI; red color: pT*α*1-DsRed2). (b) Two days after treatment of NHPDQC, length of neurite outgrowth was measured from confocal microscope images. ^∗^*P* value <0.05. (c) Total fluorescence activity for the expression of neuronal markers was analyzed in whole cell population by TissueFAXS2.0.

To monitor time-lapse changes of neuronal differentiation of MSCs by NHPDQC in vitro, we introduced a reporter plasmid DNA vector driven by the T*α*1 promoter, pT*α*1-DsRed2. Red fluorescence signals increased in the cytoplasm of MSCs treated with NHPDQC within 48 h (Figure 4(a)), compared with that of the DMSO-treated control group, suggesting that the promoter activity of the neuronal marker (T*α*1) was enhanced by NHPDQC. The neurite growth length measured on confocal microscopic images had a mean value of 53.3 ± 12.4 *μ*m for the NHPDQC-treated cells (Figure 4(b)). In contrast, in the DMSO-treated control group, the neurite length was not almost measurable meaning the lack of any morphological changes. When TissueFAX fluorescence imaging analyzer was also introduced to obtain total fluorescence signals for the whole cell population in a cell-loaded slide glass, the red fluorescence signals in pT*α*1-DsRed2-transfected MSCs after treatment of NHPDQC were approximately 10-fold higher than those in the DMSO-treated cell group (Figure 4(c)).

**Figure 5:**
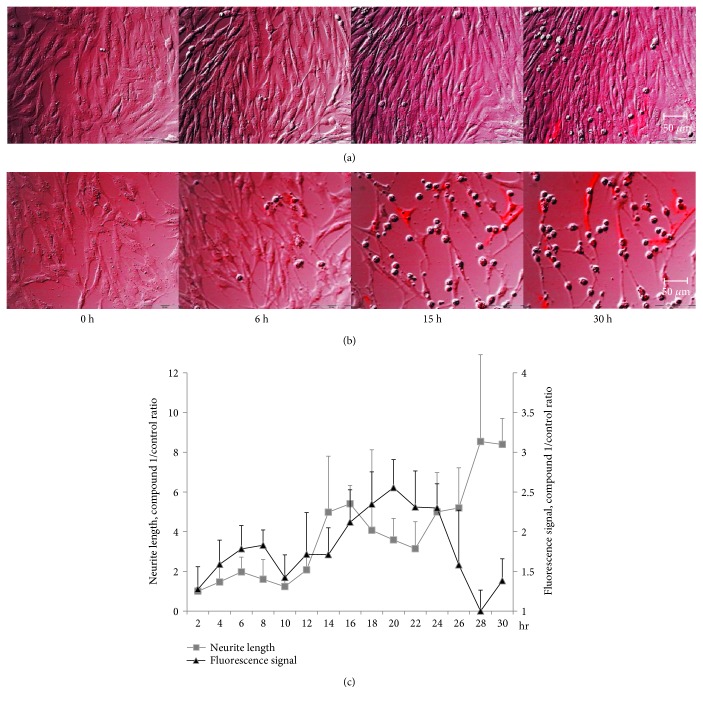
Time-lapse live cell images of (a) the DMSO-treated group and (b) the NHPDQC-treated group in pT*α*1-DsRed2-transfected MSCs. NHPDQC treatment to induce neuronal differentiation showed that the fluorescence signals in MSCs transfected with pT*α*1-DsRed2 were gradually increased according to cellular phenotypic changes. (c) Neurite growth (gray) and fluorescence signals (black) were increased in the NHPDQC-treated group than the DMSO-treated cell group from 2 h after treatment. Fluorescence signal in the NHPDQC-treated group was dropped at 24 h after treatment. In contrast, neurite growth was progressed at 30 h after treatment.

To establish the time-lapse live cell imaging system for the detection of neuronal differentiation in MSCs by NHPDQC, a live cell fluorescence microscopy equipped with CO_2_-supplied cell chamber stage was used for maintaining the live MSCs until 30 h. MSCs treated with DMSO only showed negligible T*α*1 promoter activity and no significant phenotypic alteration on time-lapse imaging (Figure 5(a)). In contrast, we observed a gradual increase in DsRed2 fluorescent signal, accompanied by morphological change in MSCs within the NHPDQC treatment group (Figure 5(b)). On the quantitative analysis of fluorescence signals, intensity in the NHPDQC-treated group increased gradually from 2 h to 24 h and then started to decrease (Figure 5(c)). However, neurite outgrowth progressed further over time in the NHPDQC-treated MSCs. This suggested that T*α*1 promoter-based reporter imaging could be used to trace fate changes of bone marrow-derived MSCs to neurons in live cell condition.

## 4. Discussion

Development of fluorescence-based evaluation system capable of tracing the neuronal differentiation of MSCs by chemical compound is crucial for examining the efficacy of neuronal differentiation of MSCs. In this study, we evaluated the neuronal transdifferentiation ability of NHPDQC for MSCs and developed in vitro monitoring system based on neuron-specific promoter-driven fluorescence reporters during neuronal differentiation of MSCs.

Quinoxaline derivatives are known to have wide range of biological properties from antimicrobial effects to anticancer effects [27]. NHPDQC was synthesized via structural modification of quinoxaline-based small molecule, and this small molecule was first identified from a chemical library to induce neurons in neuronal precursor cell lines. NHPDQC treatment in MSCs showed a significant morphology change and the increased NSE and *β*III-tubulin expression on a dose-dependent manner. Functional neuronal characteristics were also verified using electrophysiological studies, and DNA microarray analysis showed that certain cholinergic neuron receptors increased [15]. Based on these results, NHPDQC has been considered as neuronal lineage-specific inducer in MSCs.

This small molecule-based protocol for induction of transdifferentiation of MSCs into neurons may be more suitable for future clinical application than stably overexpressing neuron-inducing transcription factor because genetic modification of transplanted cells using viral vectors may induce unwelcomed side effects including innate immune response [28] and insertional mutagenesis [29]. In this study, we used a small molecule-based method to induce neuronal differentiation of rat MSCs. However, in order to accelerate clinical translation, human MSCs would be more useful and acceptable to be used for future human application. Many previous reports clearly suggest that there are similarities and differences of MSCs of different species. Human MSCs were shown to take a longer time to achieve osteogenic and chondrogenic cell differentiation phenotype, compared to the differentiation time of rat MSCs even though rat MSCs and human MSCs have a similar differentiation potential [30]. Studies evaluating transdifferentiation ability of NHPDQC in human MSCs would be necessary to take one step toward clinical application.

Many studies have reported on transdifferentiation of MSCs into neuronal lineage by treatment of chemicals such as *β*-mercaptoethanol (BME)/dimethyl sulfoxide (DMSO)/butylated hydroxyanisole or neurotrophic factors or by their coculture with neural or glial cells [31–37]. More recently, the BME-treated MSCs showed neuron-like features, expressing high level of neural-specific markers (Map2, Nefl, Tau, and nestin) [38]. However, previous neuronal induction protocol using chemicals showed relatively low efficiency and toxic effect to cultured cells [39, 40]. Also, numerous studies have focused on developing a scaffold-based neuronal differentiation induction method [39, 40]. The rGO-assembled porcine acellular dermal matrix (PADM) scaffold could promote the differentiation of MSCs into neuronal cells with high gene expression (nestin, beta tubulin-III, GFAP, and MAP2) with neurite sprouting after 7 days under neural differentiation conditions [41]. More interestingly, the exosome of differentiating neuronal cells was sufficient to induce neurogenesis of MSCs [42]. The differentiating PC12 exosome-treated MSCs showed neurite sprouting and upregulated the gene expressions of neuronal markers with a 3.5-fold higher level of miR-125b. However, the design of these 3D architectures was complicated and time consuming and took a long time to generate neuronal lineage.

In our earlier work, the estimated efficacy of NHPDQC for neuronal differentiation was more than 95% in cultured MSCs [15]. Our observation was in agreement with the previous finding, suggesting that NHPDQC would be potent neural inducer in comparison with other chemicals. Woodbury et al. [43] reported that treatment with 2% DMSO elicited morphologic change of MSCs. To eliminate the aberrant effect of nonspecific chemical treatment, we used the control group with much lower DMSO concentration. The control group without NHPDQC did not show any morphological change, indicating NHPDQC-specific induction of neuronal differentiation. Also, we introduced a more convenient method with a low-dose chemical treatment for neural transdifferentiation than that in previous studies [33, 43].

Typical methods for evaluation of neuronal differentiation include a conventional type of inverted microscope for determining cellular morphology, a confocal microscope for detecting immunophenotype, and a transmission electron microscopy for investigating ultrastructure in the fixed sample. These methods have limitation to follow up morphological change because neuronal marker expression of MSCs after committing to neuronal cells fluctuated over time. In this study, we used the neuron-specific promoter-based fluorescence reporter system for time-course tracking of neuronal differentiation in the live MSCs in vitro using time-lapse fluorescence microscope. We found that fluorescence activities in pT*α*1-DsRed2-transfected MSCs increased gradually in association with increasing neurite outgrowth after induction of neuronal differentiation by NHPDQC. High DsRed2 signals were seen clearly along the extended neurites until at least 30 h (Figure 4(b)). Because the utmost advantage of NHPDQC for inducing neuronal differentiation is high differentiation efficacy and short period time for neuronal induction (more than 95% within 48 h), transient transfection of pT*α*1-DsRed2 into MSCs was enough to monitor the progress of neuronal differentiation in MSCs.

Development of stable and safe methods for detecting serial changes of neuronal transdifferentiation would be essential in the study of in vivo transdifferentiation of MSCs. In vivo monitoring of neuronal differentiation using optical reporters was reported in living animals in many reports, by showing the change of reporter signals in vivo, using neuron-specific promoter-regulated luciferase reporters or neuron-specific miR-targeted reporters [44, 45]. Though bioluminescence reporter-based studies provide highly sensitive data with low background in vivo, this method is limited in obtaining microscopic sophisticated changes in vitro. Thus, the development of advanced multimodal imaging techniques using both luciferase and fluorescence reporters could help to better understand in vivo as well as in vitro information on transdifferentiation efficacy of MSCs.

## 5. Conclusion

In this study, we investigated the capability of a new small molecule, NHPDQC, to facilitate efficiently the neuronal transdifferentiation of MSCs. By observing the changes of fluorescent markers on time-lapse fluorescence imaging system, neuronal differentiation of MSCs could be traced temporally. This efficient method for neuronal induction using NHPDQC and the effective live cell imaging enabled tracking the efficacy of transdifferentiation of MSCs into neurons.

## Figures and Tables

**Table 1:** Information on the primers used for reverse transcription polymerase chain reaction (RT-PCR).

Target genes	Abbreviations	Nucleotide sequences	
Neuron-specific enolase	NSE	Forward Reverse	GTGGACCACATCAACAGCACTGAGCAATGTGGCGATGAG
Neuron-specific class III *β*-tubulin	*β*III-tubulin	Forward Reverse	GGCCTCCTCTCACAAGTATGTCGCCCTCTGTTAGTGC
Glial fibrillary acidic protein	GFAP	Forward Reverse	TTTCTCCTTGTCTCGAATTAGGTTTCATCTTGGAGCTTCT
Microtubule-associated protein 2	MAP2	Forward Reverse	TCGGCTCATTAACCAACCTCGAGCCACATTTGGAAGTCAC
Neurofilament medium	NF-M	Forward Reverse	GCACTAAGGAGTCCCTGGAACGCCTCGACTTTGGTCTTCTG
Synaptophysin	Synaptophysin	Forward Reverse	CCACGGACCCAGAGAACGCTGGCTGCCCGTAATC
*β*-Actin	*β*-Actin	Forward Reverse	TGGAATCCTGTGGCATCCATGAAACTAAAACGCAGCTCAGTAACAGTCCG

## Abbreviations

ALS: Amyotrophic lateral sclerosis
BM: Bone marrow
BME: *β*-Mercaptoethanol
DAPI: Dihydrochloride
DMEM: Dulbecco's modified Eagle's medium
DMSO: Dimethyl sulfoxide
FACS: Fluorescence-activated cell sorting
FBS: Fetal bovine serum
FOV: Field of view
GDNF: Glial cell line-derived neurotrophic factor
GFAP: Glial fibrillary acidic protein
MAP2: Microtubule-associated protein 2
miR: MicroRNA
MSCs: Mesenchymal stem cells
NEFL: Neurofilament, light polypeptide
NSE: Neuron-specific enolase
PADM: Porcine acellular dermal matrix
PBS: Phosphate-buffered saline
PFA: Paraformaldehyde
RFP: Red fluorescent protein
rGO: Reduced graphene oxide
RT-PCR: Reverse transcription polymerase chain reaction
T*α*1: Tubulin *α*1.
